# The Impact of Sub-valvular Apparatus Preservation on Prosthetic Valve Dysfunction During Mitral Valve Replacement

**DOI:** 10.5812/cardiovascmed.8054

**Published:** 2013-02-24

**Authors:** Alireza Alizadeh-Ghavidel, Yalda Mirmesdagh, Mehrzad Sharifi, Anita Sadeghpour, Reza Nakhaeizadeh, Gholamreza Omrani

**Affiliations:** 1 Heart Valve Disease Research Center, Rajaie Cardiovascular Medical and Research Center; Tehran University of Medical Sciences, Tehran, IR Iran; 2 Echocardiograghy Research Center, Rajaie Cardiovascular Medical and Research Center, Tehran University of Medical Sciences, Tehran, IR Iran

**Keywords:** Heart Valves, Mitral Valve, Thrombosis

## Abstract

**Background:**

Sub-valvular apparatus preservation (SAP) during mitral valve replacement (MVR) is not a new concept. Some surgeons prefer to excise the apparatus.

**Objectives:**

The aim of this study was to reduce the risk of prosthetic valve dysfunction.

**Materials and Methods:**

This retrospective study included 151 patients with the mean age of 46 years who underwent MVR (Female/male = 93/58). In the group I consisting of 39 patients, MVR with chordae preservation was performed (Bi-leaflet preservation = 20; posterior leaflet preservation = 19). In the group II consisting of 112 patients, sub-valvular apparatus was resected completely during MVR. Preoperative patients’ characteristics, including age, sex, functional status, left ventricular ejection fraction, and end-diastolic or end-systolic dimensions were statistically similar in both groups. Mean follow-up period was 60.3 ± 26 months.

**Results:**

The improvement of functional status was seen in almost all survivors but was more obvious in the group I. In early follow-up, 56.4% of group I cases and 44.1% of group II patients were classified as New York Heart Association class I. These rates were 84.2% and 71.2% in mid-term follow-up, respectively (P < 0.001). Mortality rate was significantly lower in the group I (2.6%) compared to the group II (8.9%) (P = 0.03). There was a trend for higher frequency of postoperative atrial fibrillation in the group II compared to that in the group I (52.7% vs. 38.5%, P = 0.12).The incidence of prosthetic valve dysfunction (PVD) was 5.1% in the group I and 4.5% in the group II, but this difference was not statistically significant (P = 0.56).

**Conclusions:**

Preservation of mitral annulus and papillary muscle continuity may enhance post- MVR cardiac performance with low mortality and morbidity rates. The risk of PVD was not significantly higher than conventional MVR in our series.

## 1. Background

In the past years, despite major advances in surgical techniques, anesthesia, myocardial protection, and postoperative care, there was not a substantial reduction in the morbidity and mortality associated with mitral valve replacement (MVR) (1). Left ventricular performance is better after mitral valve repair than that after MVR, which is attributed to the preservation of sub-valvular apparatus of mitral valve (2). Although current evidence has shown that repair of mitral valve is superior to its replacement, in some cases of rheumatic and degenerative valve diseases causing valvular disorganization, the valve is not suitable for repair. Also, repair of mitral valve may not be feasible in some circumstances due to low experience of the surgeon or unavailability of trans-esophageal echocardiography (TEE). In these cases, valve replacement is the only solution (3). Some authors advocated the valve sparing operation both for reducing operative mortality and postoperative morbidity. Their primary purpose is to leave the continuity between mitral annulus and papillary muscles intact (4, 5).

The sub-valvular apparatus consists of left ventricular free wall, two papillary muscles, and the chordae tendineae. During ventricular systole, the sub-valvular apparatus prevents the mitral leaflets from prolapsing into the left atrium. Papillary muscles and chordae tendineae also cause effective left ventricular contraction through a process known as “annulo-ventricular continuity”. According to this process, left ventricular geometry and function depend on dynamic relationship between left ventricular wall and mitral valve annulus. As the papillary muscles contract during the isometric phase of cardiac cycle, the closed mitral valve is brought down into left ventricle resulting in a reduction in longitudinal axis and an increase in short axis (6). This causes increased myocardial fiber stretch that generates greater tension, contraction, and stroke volume.

The concept of SAP is presented more than 40 years ago (7). Frequent techniques were reported to preserve sub-valvular apparatus for morbidity and mortality reduction following mitral valve replacement. Although several studies demonstrated that isolated posterior leaflet preservation did not improve left ventricular (LV) performance (6), anterior leaflet had critical role for LV function by preserving the geometry of left ventricle (8). Renewed interest in MVR chordal-sparing techniques was -motivated by the report of Miller et al. (9) stating that operative survival was enhanced by SAP due to the decreased risk of ventricular rupture. Despite further reports of improved long-term survival following MVR with chordal preservation (10), to date, this effect was not systematically assessed nor the technique translated into a standard clinical practice.

## 2. Objectives

The aim of this study was to reduce the risk of prosthetic valve dysfunction.

## 3. Materials and Methods

In this retrospective cohort study, medical records of 151consecutive patients (mean age 46 ± 14 years) who underwent MVR from January 1996 to November 2006 at Rajaie cardiovascular medical and research center (Tehran, Iran) were reviewed. The patients were subdivided into two groups: group I, the patients who underwent MVR with sub-valvular apparatus preservation (SAP) technique (39 patients), and group II: the patients who underwent conventional MVR with total excision of native valve and sub-valvular apparatus (112 patients).The surgical technique was performed based on the surgeon's preference. The cardiac rhythm was analyzed on ordinary 12 –lead electrocardiography. In 15 patients (38.5%) of group I and 62 patients (55.9%) of group II, atrial fibrillation was manifested. Cardiac catheterization was performed for all patients aged more than 40 years.

Two study groups were matched well regarding preoperative patients characteristics (Table 1). 

**Table 1.** Preoperative Patients’ Characteristics

	MVR ^a^ Groups		
	SAP ^a^ (n = 39)	VR ^a^ (n = 112)	P value
**Number of Patients**			0.68
Male	14	44	
Female	25	68	
**Age, y**	42 ± 18	47 ± 13	0.61
**BSA**	1.7 ± 0.2	1.6 ± 0.2	0.76
**Pre-op NYHA ^a^**	2.23 ± 0.2	2.38 ± 0.2	0.53
**Pre-op LVEDD ^a^, (mm)**	5.5 ± 0.9	5.2 ± 0.8	0.32
**Pre-op LVESD ^a^, (mm)**	4 ± 0.8	3.6 ± 0.7	0.44
**Pre-op LVEF ^a^**	52 ± 8.9	51 ± 8.8	0.59
**Trans M.V Mean Gradient (mmHg)**	10 ± 5	17 ± 6	0.02
**Follow up, Month**	55 ± 28	62 ± 25	0.45
**Mitral Valve pathology**			0.08
MS ^a^	24 (61.5%)	85 (76.6%)	
Isolated MR ^a^	15 (38.5%)	27 (23.4%)	
**Preoperative AS**	15 (38.5%)	62 (55.3%)	0.13
**Significant CAD ^a^**	1 (2.6%)	5 (4.4%)	0.11

^a^ Abbreviations: AF: Atrial Fibrallation, BSA: Body Surface Area, CAD: Coronary Artery Disease, G: Gradient, LVEDD: Left Ventricular End-diastolic Diameter, LVEF: Left Ventricular Ejection Fraction, LVESD: Left Ventricular End-systolic Diameter, MR: Mitral Regurgitation, MS: Mitral Stenosis, MV: Mitral Valve, MVR: Mitral Valve Replacement, NYHA: New York Heart Association, SAP: Subvalvular Apparatus Preservation, VR: Valve Replacement

The pre-op and post-op functional capacity (New York Heart Association; NYHA classification) were assigned by either consulting cardiologist or operating surgeon. In-hospital mortality was defined as death during the first 30 days from operation. All patients were followed post operatively by physical exams, ECG, trans-thoracic echocardiography (TTE), or TEE (if indicated) at least for 6 months. Mean follow-up period was 60.3 ± 26 months (Range: 6-120 months).

### 3.1. Surgical Technique

All surgeries were performed through a standard median sternotomy under general anesthesia. Cardiopulmonary bypass (CPB) was established via ascending aortic as well as bicaval venous cannulation. The hematocrit was maintained between 20% and 25%, pump flow rates between 2.0 and 2.5 L/min/m2, and mean arterial pressure between 50 and 70 mmHg during CPB.

Myocardial protection consisted of cold antegrade crystalloid cardioplegia in the vast majority of cases, and warm antegrade cardioplegia in the minority. Cardioplegic solution was St-Thomas II at the dose of 15cc/kg and repeated half-doses every 20-30 minutes. Topical cooling was performed using ice slush solutions. Retrograde cardioplegia was used only in patients with severe coronary artery disease. Moderate systemic hypothermia (28 ºC to 32 ºC) was employed during CPB for all cases. The mitral valve was approached through an incision in the left atrium, just posterior to the inter-atrial groove. The decision of whether or not to preserve sub-valvular apparatus was made intra-operatively and at the discretion of attending surgeon according to valve pathology (rheumatic or degenerative), degree of chorda shortening, calcification, thickness, and pliability of leaflet.

In those patients that the surgeons preferred to perform sub-valvular preservation, it was attempted to retain native sub-valvular tissue whenever possible. Redundant valvular leaflet tissue was imbricated between the annulus and prosthetic sewing ring, or excised if necessary. In both groups, three stay sutures (2-0 pledgeted ethibond) at hours 4, 8, 12 (four sutures in wide anterior leaflet at hours 2, 4, 8, 10) were applied using 2-0 prolene running suture, the prosthetic valve (ST Jude in 128 cases, CarboMedics in 21 cases, Bioprosthesis in 2 cases) was implanted in the annulus in a way that all suture bites passed through mitral annulus and the leaflet tissues were folded between prosthesis sewing ring and annulus. No additional plication suture was used.

### 3.2. Statistical Analysis

Statistical analyses were performed using SPSS 15 for Windows (SPSS Inc., Chicago, Illinois). Clinical data were expressed as mean values ± standard deviation. Differences were analyzed using paired and independent Student’s t-test, Chi-square test, and Fisher’s exact test. Pre-operative and post-operative values within the same group were compared using paired samples Student’s t-test. Pre-operative and post-operative values in different groups were compared using independent samples Student’s t-test. A value of P < 0.05 was considered statistically significant.

## 4. Results

Among 151 patients, 39 cases (25.8%) were in the SAP group (group I) of which 20 patients (13.2%) underwent MVR with preservation of both leaflets and 19 patients (12.5%) with preservation of only posterior leaflet. 109 patients (72%) suffered from dominant mitral stenosis (MS) and 42 patients (28%) from dominant mitral regurgitation (MR). 112 patients (74.1%) underwent conventional MVR with valve resection (VR group, or group II). Mitral valve pathology was MS ± MR in 24 patients (61.5%) of group I vs. 85 patients (76.6%) of group II. Isolated MR was the main pathology in 15 patients (38.5%) of group I vs. 27 patients (23.4%) of group II (P = 0.08). Patients in VR group exhibited significantly higher mean diastolic mitral gradients (17 ± 6 vs. 10 ± 5, P = 0.02).This difference was attributed to more cases with MS in this group and unavailability of valves with proper size for replacement; in addition, preservation of both leaflets was not feasible.

The improvement of functional status was observed in almost all survivors but was more obvious in the group I. Early follow-up was considered 1 month from surgery. In late follow -up, 84.2% and 71.2% of the patients of group I and group II were classified as NYHA class I, respectively (P < 0.001). The mean NYHA class was 1.4 ± 0.5 and 1.5 ± 0.5 for group I group II, respectively (P = 0.66). The post-op incidence of atrial fibrillation (AF) rhythm was 38.5% (15 cases) in the group I and 52.7% (59 cases) in the group II (P = 0.12). The amount of bleeding within the first 24 hours of operation was 456 ± 331cc in the group I and 458 ± 389cc in the group II (P = 0.81). High dose inotrope support required for 3 patients (7.7%) of group I but for 14 patients (12.6%) of group II (P = 0.04). The only significant difference observed in post-op complications between study groups was the incidence of myocardial failure which occurred in 3 cases (7.7%) of group I and 14 cases (12.6%) of group II (P = 0.02). Compared to the group II, some complications, including respiratory failure, renal failure, deep sternal infection, ventricular arrhythmia, prosthetic valve endocarditis, and atrio-ventricular (AV) groove rupture were absent in the group I. However, this was not statistically significant. Cerebrovascular accident (CVA) was seen in 1 patient (2.8%) of group I, but not seen in the group II. Again, this was not statistically significant. Table 2 shows early post-op results.

**Table 2.** Early postoperative Results

	MVR ^a^ Groups		
	SAP ^a^ (n = 39)	VR ^a^ (n = 112)	P value
**AF ^a^ Rhythm**	15 (38.5%)	59(52.7%)	0.12
**Bleeding, ml**	456 ± 331	458+/-389	0.81
**High dose Inotropic support**	3 (7.7%)	14 (12.6%)	0.04
**Complications**			
Respiratory failure	0	2 (1.8%)	0.56
Myocardial failure	3 (7.7%)	14 (12.6%)	0.02
Renal Failure	0	2 (1.8%)	0.44
CVA ^a^	1 (2.6%)	0	0.79
Deep Sternal infection	0	2 (1.8%)	0.27
Ventricular Arythmia	0	3 (2.7%)	0.53
PVE ^a^	0	2 (1.8%)	0.54
AV a groove rupture	0	1 (0.9%)	0.39
Death	1(2.6%)	10 (8.9%)	0.03

^a^ Abbreviations: AF: Atrial Fibrillation, AV: Atrioventricular, CVA: Cerebrovascular Accident, PVE: Prosthetic Valve Endocarditis, SAP: Subvalvular Apparatus Preservation, VR: Valve Replacement

Overall mortality rate was 8.6%. Overall in-hospital death was 11 cases (7.28%); one (2.6%) in the group I, and 10 (8.9%) in the group II (P = 0.039). There was no late mortality in the group I, but two patients (19%) of group II died during post-op follow-up (P = 0.18). The most common causes of death in our study were myocardial failure (4 patients), excessive bleeding with disseminated intravascular coagulation (DIC), (2 cases), and pulmonary insufficiency (2 cases). The rest of deaths were due to acute renal failure (1 case), mediastinitis (multiple organ dysfunction syndrome; MODS) (1case), and AV groove rupture (1 case). There were not any significant differences between mentioned causes of death (P = 0.10). As previously stated, late mortality occurred in 2 patients, one due to the congestive heart failure (CHF), but for the other patient, no specific etiology was detected. The incidence of prosthetic valve dysfunction (PVD) was 5.1% (2 cases) in the group I vs. 4.5% (5 cases) in the group II, but this difference was not considered statistically significant (P = 0.56). Table 3 shows the characteristics of patients with PVD. 

**Table 3.** Characteristics of Patients with Prosthetic Valve Dysfunction

	MVR ^a^ Groups		
	SAP ^a^ (n = 39)	VR ^a^ (n = 112)	P value
**Age, yrs**	33.3 ± 13	30 ± 7	0.28
**Sex (female)**	22 (66.7%)	67 (60%)	0.96
**AF ^a^ Rhythm**	8 (20%)	75 (66.7%)	0.011
**Prosthesis Size**	27.1 ± 1	28.6 ± 1	0.74
**Sever LV ^a^ Dysfunction**	0	0	P > 0.99
**Previous Valve Surgery**	0	0	P > 0.99t
**Concomitant Procedure**	0	2 (2%)	0.69
**Mean Time until Malfunction**	41 ± 26	39 ± 15	0.88
**Incidence of Valve Dysfunction**			0.56
Mechanical Problem	0	0	
Thrombosis (Poor Anticoagulation Therapy)	1 (3%)	3 (3%)	
Tissue Ingrowth	1 (3%)	2 (2%)	

^a^ Abbreviations: AF: Atrial Fibrillation, LV: Left Ventricle, SAP: Subvalvular Apparatus Preservation, VR: Valve Replacement

Mean age of patients with PVD was 33.3 years in the group I and 30 years in the group II. In both groups, dysfunction was more prevalent in female; 66.7% of patients with PVD in the group I and 60% in the group II were women. None of the patients with PVD had severe LV dysfunction or previous valve surgery. Mean time until malfunction was 41 ± 26 months in the group I and 30 ± 15 months in the group II (P = 0.88). The cause of valve dysfunction was thrombosis in 4 cases; 1 in the group I, and 3 in the group II. None of the PVD observed was due to mechanical problem of prosthetic valve. Tissue in-growth or pannus formation caused PVD for the remaining cases: 1 in the group I, and 2 in the group II. In patients with PVD, AF rhythm was more prominent in the group II (66.7%) compared to the group I (20%) (P = 0.011). Table 4 shows operation data. 

**Table 4.** Operation data

	**MVR ^a^ Groups**			
	SAP ^a^ (n = 39)	VR ^a^ (n = 112)	P value
**Valve size**	28.5 ± 1.2	28.9 ± 1.2	0.78
**Valve type**			0.92
Mechanical	39 (100%)	110 (98.2%)	
Bioprosthesis	0	2 (1.8%)	
**AOX ^a^**	34.6 ± 14	46.3 ± 22	0.07
**CPB ^a^**	57 ± 23	73.7 ± 37	0.06
**Concomitant Procedure**			0.71
None	36 (92.3%)	87(76.5%)	
CABG ^a^	1 (2.6%)	5 (4.5%)	
AVR ^a^	2 (5.1%)	15 (13.5%)	
TVR ^a^ or TV a repair	0	3 (2.7%)	
AVR ^a^ +TVR a	0	1 (0.9%)	
ASD ^a^	0	1 (0.9%)	

^a^ Abbreviations: AOX: Aortic Cross Clamping, ASD: Atrial Septum Defect, AVR: Aortic Valve Replacement, CABG: Coronary Artery Bypass Grafting, CPB: Cardio Pulmonary Bypass, SAP: Subvalvular Apparatus Preservation, TV: Tricuspid Valve, TVR: Tricuspid Valve Replacement, VR: Valve Replacement

In our study, there were no statistically significant differences between the two groups with respect to the aortic cross-clamp (AOX) and CPB time. Mean time for AOX was 34.6 min. in the group I, and 46.3 min. in the group II (P = 0.07). For CPB time, these figures were 57 min. vs. 73 min. for group I and group II, respectively (P = 0.06).

## 5. Discussion

After the reports of favorable results with mitral valvuloplasty for patients with chronic mitral insufficiency, mitral valve replacement with preservation of the chordae tendineae was revised by David et al. (11). These authors reported convincing clinical evidence, favoring the maintenance of the annulo-papillary continuity, which had already been demonstrated in the pioneering study by Lillehei et al. (12). Subsequent years brought clinical and experimental evidence supporting this concept, and propagate the recommendation of not-to- excise all chordae tendineae in MVR. 

The main cause of death after MVR is myocardial failure (13). Several animal and human echocardiographic physiological studies have shown better maintenance of left ventricular function following SAP (7, 13). It has been suggested that the reason is importance of papillary muscles for left ventricular contraction as they pull mitral ring toward the apex, causing shortening of longitudinal axis and providing more sphericity for the chamber, which result in better blood ejection (14). A surgical mortality ranging from zero to 9.5% for the groups undergoing MVR with chordal preservation has been reported (15, 16). In-hospital death in our study was significantly lower in SAP group (P = 0.039). The most common cause of death in our investigation was myocardial failure, too, which comprised 38.4% of of deaths among MVR patients in both groups. Risk factors for mortality in our study included post-op use of inotropic agents (P = 0.009), previous mitral valve surgery (P = 0.03), and pre-op NYHA class III (P = 0.02). Factors of age, sex, CPB time, AF rhythm, valve size, valve pathology, concomitant procedures, and type of surgery were not prognostic for mortality. Similarly, Lee and associates examined and found that failure to preserve the sub-valvular apparatus was an independent predictor of early and late mortalities (15). Wu and associates also demonstrated improved early survival and LV function in the preservation group (16). The reduced inotropic use and peri-operative mortality rate associated with SAP demonstrated in this study, had significant implications for the management of MVR patients being at higher risk such as the elderly, patients with dilated cardiomyopathy, multiple valve disease, those undergoing re-do surgery, or patients with severely impaired left ventricular function. In this group, employing preservative techniques could potentially reduce their operative mortality. In addition to the beneficial effects of MVR with sub-valvular preservation on LV performance, this technique likely decreases the risk of myocardial rupture, which is an uncommon but catastrophic complication of MVR (17). In our study, AV groove rupture was observed in only 0.9% of patients of VR group. In preserving the sub-valvular apparatus and annulo-ventricular continuity, three main concepts must be appreciated:

1. Valve tissue preservation to reduce the risk of ventricular rupture.
2. Preservation of mitral annulus symmetry to allow better contact between the valve prosthesis and mitral annulus and avoid from possible consequent paravalvular leak.
3. Preservation of natural chordae tension to allow more physiological systolic and diastolic functions of left ventricular.

The incidence of prosthetic valve dysfunction (PVD) in our series was slightly higher than that in SAP group (P = 0.174). According to our multivariate analysis, surgical method, pre- or post-op AF rhythm, pre- or post-op NYHA, pre- or post-op EF, age, sex, valve size, valve type, valve pathology, body surface area BSA, and previous mitral surgery were not risk factors for PVD. Despite existing evidence suggesting that SAP reduces morbidity and mortality rates (9), the sub-valvular apparatus is not always possible to be preserved. It is argued that the preserved sub-valvular apparatus prevent an adequately sized prosthetic valve from being used and cause left ventricular outflow tract obstruction by interfering with prosthetic valve function (18). Whilst techniques to eliminate outflow tract obstruction following sub-valvular apparatus preservation have been described (19), it is reported that some of the preservation techniques cause alteration of the left ventricular geometry, causing rupture of the papillary muscles, systemic embolization, and dehiscence of the mitral leaflets from their transposed position, as well as, increasing the ischemic time (14, 18).

Left ventricular outflow tract (LVOT) obstruction is the main complication of bi-leaflet preserving operation reported in the recent years (4, 5). Redundant chordae tendineae, reduction of left ventricle size after surgery, and excessive mitral anterior leaflet tissue may cause LVOT obstruction. In our study, prosthetic valve size was not significantly different between the two groups (P = 0.78). Likewise, no LVOT obstruction was noted in our series. Another dilemma in this topic is patients with mitral stenosis. There have been some limitations and points of concern regarding chordae preservation in MVR for mitral stenosis. Firstly, it is not possible to preserve mitral apparatus in every patient. SAP can be difficult in the presence of calcified, rigid leaflets, which are especially common in patients with rheumatic valve disease. Secondly, in patients with mitral stenosis, the sub-valvular apparatus is diseased due to thickening and fusion, creating constrain in the function of the left ventricle. The efficacy of SAP in this group of patients is reduced compared to mitral regurgitation (20).

Thirdly, in our series the most common pathology was MS ± MR in both groups; 61.5% in SAP group and 76.6% in VR group, but as noted earlier, the mitral valve pathology was not a risk factor for PVD or mortality,; so if the valve is suitable, the technique of MVR with preservation of chordae tendineae seems to have a beneficial effect on post-operative left ventricular performance in patients with mitral stenosis (21). Fourthly, re-do MVR patients comprise a high-risk group, who deserve further attention. Preservation of sub-valvular apparatus is associated with a decreased risk of mortality during re-do MVR. Although sub-valvular preservation may increase the complexity of the operation, the use of this technique is strongly recommended in first-time and re-do MVR surgeries (22).

It is noteworthy that over one-half of the patients in the current study did not receive any form of sub-valvular preservation during MVR. There are two probable explanations for this finding. Firstly, the beneficial effects of sub-valvular preservation were still controversial during the time of current study, particularly in the earlier years. Secondly, preservation of the sub-valvular apparatus may increase the complexity of operation, which might force some surgeons to avoid this technique, and makes some others to believe that the preservation of sub-valvular apparatus may increase the risk of further PVD. It should be re-emphasized that the decision of whether or not to preserve the sub-valvular apparatus was at the discretion of operating surgeon in the current study. Preoperatively treated bacterial endocarditis was also another dilemma for the leaflet-preserving operations. We did not perform SAP after bacterial endocarditis and we still hesitate to perform bi-leaflet-preserving operations on these patients. We think that it should be clarified in the future studies.

It is important to address certain areas with regards to SAP. Firstly, primary data on long-term quality of life after MVR is needed. Secondly, further research is clearly needed to compare bi-leaflet versus posterior leaflet preservation techniques. Further work is also required to investigate different subgroups of patients with mitral regurgitation due to varying causes (ischemic disease, re-do surgery, or degenerative disease). Nowadays, although, the first choice of treatment for degenerative mitral valve diseases or functional MR is to repair the valve instead of replacing, we have to keep this aspect in mind that the process of MV repair may not be feasible for all cases. In addition, the result of the repair may not be satisfying and significant residual MR necessitates replacing the valve. In such condition it seems that MVR with sub-valvular apparatus preservation is the best alternative for these patients. The present study was a retrospective review of surgical intervention in patients with mitral valve pathology. Therefore, we also have to consider this aspect that retrospective and nonrandomized nature of the study were limitations for this study.

This study supports previous evidence of the superior efficacy of SAP compared to conventional MVR. Surgeons should be familiar with a number of preservation techniques as the current evidence suggests that early and long-term clinical outcome can be improved by maintaining ventricular-mitral continuity. The results support previously published data, demonstrating that survival is significantly improved following SAP, and represents further evidence that, whenever technically feasible, SAP should be routinely performed. We believe that MVR with SAP is the best alternative to mitral valve repair in degenerative or ischemic MR and a good option for valve replacement in rheumatic disease (at least posterior leaflet preservation), when feasible.
